# Evolutionarily-Related *Helicobacter pylori* Genotypes and Gastric Intraepithelial Neoplasia in a High-Risk Area of Northern Italy

**DOI:** 10.3390/microorganisms8030324

**Published:** 2020-02-26

**Authors:** Sonia Toracchio, Rosario Alberto Caruso, Silvia Perconti, Luciana Rigoli, Enrico Betri, Matteo Neri, Fabio Verginelli, Renato Mariani-Costantini

**Affiliations:** 1 Center for Advanced Studies (CAST/CeSI-MeT), “G. d’Annunzio” University of Chieti-Pescara, 66100 Chieti, Italy; sonia.toracchio@gmail.com (S.T.); percontisilvia@gmail.com (S.P.); mneri@unich.it (M.N.); 2 Department of Human Pathology in Adult and Developmental Age “Gaetano Barresi”, Section of Pathology, University of Messina, 98123 Messina, Italy; rocaruso@unime.it (R.A.C.); lrigoli@unime.it (L.R.); 3 Department of Medical, Oral and Biotechnological Sciences, “G. d’Annunzio” University of Chieti-Pescara, 66100 Chieti, Italy; 4 Department of Pathology, Istituti Ospitalieri, 26100 Cremona, Italy; enrico.betri@alice.it; 5 Department of Medicine and Aging Science, “G. d’Annunzio” University of Chieti-Pescara, 66100 Chieti, Italy; 6 Department of Pharmacy, G. d’Annunzio University of Chieti-Pescara, 66100 Chieti, Italy

**Keywords:** *Helicobacter pylori*, virulence factors, pathogenicity, phylogenetic diversity, gastric intraepithelial neoplasia, Italy

## Abstract

*Helicobacter pylori* (*Hp*) is the major recognized risk factor for non-cardia gastric cancer (GC), but only a fraction of infected subjects develop GC, thus GC risk might reflect other genetic/environmental cofactors and/or differences in virulence among infectious *Hp* strains. Focusing on a high GC risk area of Northern Italy (Cremona, Lombardy) and using archived paraffin-embedded biopsies, we investigated the associations between the *Hp vacA* and *cagA* genotype variants and gastric intraepithelial neoplasia (GIN, 33 cases) versus non-neoplastic gastroduodenal lesions (NNGDLs, 37 cases). The *glmM* gene and the *cagA* and *vacA* (s and m) genotypes were determined by polymerase chain reaction (PCR) and sequencing. *Hp* was confirmed in 37/37 (100%) NNGDLs and detected in 9/33 GINs (27%), consistently with the well-known *Hp* loss in GC. *CagA* was detected in 4/9 *Hp*-positive GINs and in 29/37 NNGDLs. The *vacA* s1a and m1 subtypes were more common in GINs than in NNGDLs (6/7 vs. 12/34, p=0.014, for s1a; 7/7 vs. 18/34, p=0.020 for m1), with significant *vacA* s genotype-specific variance. The GIN-associated *vacA* s1a sequences clustered together, suggesting that aggressive *Hp* strains from a unique founder contribute to GC in the high-risk area studied.

## 1. Introduction

*Helicobacter pylori* (*H. pylori*, *Hp*) infection is an ascertained major risk factor for gastric and duodenal ulcer, non-cardia gastric cancer (GC), and gastric mucosa-associated lymphoid tissue lymphoma [1,2,3]. However, these pathological processes depend on a number of variables, including *Hp* virulence factors, host susceptibility, and environmental cofactors [3,4]. Focus on the pathogen resulted in the identification of specific virulence markers [5,6]. The cytotoxin-associated gene (*cagA*), not present in every *Hp* strain, is a marker for a genomic pathogenicity island associated with *Hp* infection severity [5,7]. The vacuolating cytotoxin A (*vacA*) gene, present in all *Hp* strains, encodes a protein that directly induces progressive vacuolization, mitochondrial damage, cytochrome c release, and cell death [8,9]. Two variable parts, the signal (s) and the middle (m) regions, can be distinguished within *vacA*, defining specific *vacA* genotypes that, in various populations, are associated with disease severity [10]. The *Hp* strains possessing the *vacA* s1 genotype are highly cytotoxic and have been linked to peptic ulcer and GC [5,11]. Italy is a country with high *Hp* infection rates, ranging from 40% to 71%, and with a high burden of antibiotic-resistant *Hp* strains [12,13,14,15]. However, GC incidence is higher in definite regions of Northern and Central Italy compared to Southern Italy and the Italian islands [16,17,18], despite the lower *Hp* infection prevalence in Northern and Central Italy [19]. It is still debated whether the higher geographic risk of GC in Northern and Central Italy is due to host, environmental, or bacterial factors or to combinations among such factors [20,21,22,23,24,25,26,27]. Here, to assess the relationships between *Hp* virulence factors and non-cardia gastric intraepithelial neoplasia (GIN), we analyze the *Hp* genotypes found in an archived paraffin-embedded series of GINs and non-neoplastic gastro-duodenal lesions (NNGDLs) from Cremona (Lombardy), a high GC risk area in Northern Italy [16,28].

## 2. Materials and Methods 

### 2.1. Cases and Histopathological Evaluation

Archived formalin-fixed, paraffin-embedded endoscopic or surgical biopsy specimens from a total of 70 patients with GIN or non-neoplastic gastro-duodenal lesions (NNGDL) were selected after a pathological review from the 1998-1999 files of the Pathology Unit, Cremona Hospital, Cremona, Lombardy (a well-known high-GC-risk region in Italy [16,28]). These included 33 biopsy samples representative of non-cardia GIN, of which 12 were from patients also diagnosed with invasive GC (mean age of patients 66 years, range 50–82 years, 11 females, 22 males) and 37 biopsy samples representative of the NNDGL (mean age of patients 52 years, range 29–75 years, 16 females, 21 males). The 37 NNDGL biopsies presented antral-predominant non-atrophic *Hp*-positive gastritis that has a low risk of gastric carcinoma [29], associated with gastric erosions in 17 cases and with duodenal ulcer in 10 cases.

Histological diagnosis of GIN was restricted to the biopsies showing both altered glandular architecture and abnormalities in cytology and differentiation but lacking any (even doubtful) infiltrating features [30,31]. For diagnostic purposes, the NNGDLs were documented by two biopsies from the antrum, one from the angulus, two from the corpus, and two from the duodenal bulb. For each biopsy, four-micrometer sections stained with hematoxylin-eosin (histological examination) and with Giemsa (*Hp* identification) were re-reviewed by one of us (R.A.C.) according to the Updated Sydney System [32]. Only the antral biopsies, which showed patent inflammatory changes and were *Hp*-positive by Giemsa staining, were used for *Hp* DNA analysis.

The study was performed in agreement with the guidelines of the Declaration of Helsinki for human medical research. The anonymized archived paraffin-embedded tissue blocks exceeded the 20 years-time limit requiring an ethics approval for research use, according to Italian regulations [33].

### 2.2. DNA Extraction, Polymerase Chain Reaction (PCR) and Sequence Analyses

DNA extraction from formalin-fixed, paraffin-embedded biopsies was as previously reported [34]. Amplifications were carried out using nested or semi-nested PCRs targeting short amplicons retrievable from damaged DNA (Table S1) [35]. PCR specificity, confirmed by sequencing, was tested on 3 clinical *Hp* isolates and 3 *Hp*-negative human DNAs (not shown). To evaluate assay sensitivity, the DNAs from the clinical isolates were quantified by UV spectrophotometry and used to prepare 10-fold serial dilutions ranging from 10 ng to 1 fg. Positive amplifications were obtained with as little as 10 fg of DNA (not shown).

GIN and NNGDL DNAs were first amplified for the human *β-globin* sequence, to assess DNA quality, then the GINs, for which the *Hp* status was not documented by Giemsa staining, were tested for the *glmM* (*ureC*) gene [35] to obtain molecular evidence of *Hp* infection. Next, the *glmM*-positive GINs and all the Giemsa-positive NNGDLs were amplified for the conserved *cagA* 5’-region and for *vacA* s and m. PCR products were directly sequenced using an ABI PRISM BigDye^TM^ Terminator v3.1 Cycle Sequencing Ready Reaction Kit (Applied Biosystems, Foster City, CA, USA) and sequence variants were confirmed on independent DNA amplifications. Sequences were deposited in the GenBank under accession numbers from EU881365 to EU881408 (*vacA* s, 43 sequences), EU881409 to EU881456 (*vacA* m, 47 sequences), and EU881457 to EU881492 (*cagA*, 35 sequences).

### 2.3. Data Analysis

Phylogenetic trees and multidimensional scaling (MDS) plots were constructed using the *Hp* genotypes detected in the present study and 55 GenBank *Hp* genotypes of various geographic origin characterized for the *vacA* s and m regions. These included 10 genotypes from Colombia, 4 from the USA (unspecified States), 9 from Alaska, 2 from Arizona, 3 from Kenya, 1 from Italy, 1 from the United Kingdom, 2 from Kazakhstan, 15 from Japan, 3 from Thailand, 1 from Korea, 1 from Taiwan, 1 from China, and 2 geographically undefined. Comparison of *vacA* sequences was performed for nucleotide positions 252-330 (*vacA* s, 79 bp) and 2393-2496 (*vacA* m, 104 bp), using GenBank accession number #S72494 as reference. The phylogenetic tree was constructed through the neighbour-joining method using MEGA X [36]. The Kimura 2-parameter [37], with a uniform rate of heterogeneity, was applied as a genetic distance measure to calculate the distance matrix. Significance of clusters was calculated using bootstrap percentages after replication of 10,000 trees. The genetic distances for *vacA* s and m across 100 *Hp* sequences (45 from this study and 55 from GenBank) were graphically represented on a two-dimensional space using MDS (SPSS for Windows, version 11.5).

The F-statistic (Fst) approach [38] was applied to verify statistical differences in genotype distributions. Analysis of molecular variance (AMOVA), as implemented in Arlequin suite v3.5 [39], was carried out to analyze the frequencies of *vacA* s, *vacA* m, and combined *vacA* s and m genotypes. Matrixes of pairwise Fst values were plotted using the MDS procedure. Significance was assessed by 10,000 permutations.

To obtain median-joining (MJ) networks of *vacA* s sequences, the DnaSP v.6 software [40] was applied to the sequence data to identify segregating variants. Tables of variants were used in the MJ algorithm [41] option of NETWORK 5 (Fluxus Technology Ltd).

Fu’s Fs-test of selective neutrality [42], which detects whether the pattern of diversity in a population is consistent with neutrality, was performed using Arlequin suite v3.5. If the test significantly deviates from neutral expectation, it is assumable that selection and/or recent population expansion is/are responsible for the observed diversity pattern. Since Fs tends to be negative when there is an excess of recent mutations (and therefore an excess of rare genotypes), a large negative Fs value can be taken as evidence against the neutrality of mutations.

The network clade subclustering structure was verified with the Maximum Parsimony (MP) method [43] using MEGA X [36]. The bootstrap consensus tree, inferred from 1000 replicates, was taken to represent the evolutionary history of the *Hp* genotypes in the considered network clade [44]. The MP tree was obtained using the Close-Neighbor-Interchange algorithm with search level 3 [43,45], in which initial trees were generated with random addition of sequences (100 replicates). Risk analysis was assessed calculating odds ratios (OR) and confidence intervals (CI) from two-by-two tables.

To investigate the phylogenetic relationships between our isolates and previously characterized *Hp* strains, the *cagA* 5’-region sequences were aligned with CLUSTAL W [46]. A phylogenetic tree was then constructed through the neighbor-joining method using MEGA3 version 3.0. Based on the presence of *cagA*, 36/55 GenBank sequences were included in this analysis.

## 3. Results

### 3.1. GIN-Associated Hp Genotypes

The 33 GINs, whose *Hp* status could not be assessed by low-sensitivity Giemsa staining due to the well-known loss of *Hp* during gastric carcinogenesis [3], were first tested for the *glmM* gene sequence. The *glmM*-positive samples were then analyzed for the *cagA* and *vacA* genes (s and m regions) (Table S2). Overall, 9/33 GINs (27%) were *glmM*-positive and 4 of these were *cagA*-positive. The lack of *cagA* in some GINs is consistent with the evidence that *cagA* may contribute to gastric carcinogenesis but is not required for the maintenance of the neoplastic phenotype after transformation [47]. One of the 4 *cagA*-positive GINs (Italy 7, Table S2) yielded 2 *cagA* sequences differing at np 15012 (GenBank #AF282853), suggesting double infection. Both the *vacA* s and m regions were amplified in 6/9 *glmM*-positive GINs. Of the remaining 3 *glmM*-positive cases, one (Italy 9) was not amplifiable for both *vacA* regions, while the other two were not amplifiable for *vacA* m (Italy 2) and for *vacA* s (Italy 8), respectively. All the *Hp* strains identified in GINs presented the s1 genotype, corresponding to the s1a subtype in 6/7 cases. Notably, 4 cases shared the same *vacA* s and m sequences. The remaining *vacA* s strain (Italy 5) matched subtype s1b, except for Ala18, typical of s1a, suggesting recombination between s1a and s1b. The *vacA* m1 type was detected in all cases.

### 3.2. NNGDL-Associated Hp Genotypes

The 37 *Hp*-positive NNGDLs were directly analyzed for the *cagA* and *vacA* genes. Twenty-nine cases (29/37, 78%) were *cagA*-positive, including 7 with multiple *cagA* genotypes (Table S2).

The *vacA* s sequence could be typed in 34/37 cases, of which 29 (29/34, 85%) were s1 and 5 (5/34, 15%) were s2 (Table S2). Of the 3 cases not amplifiable for *vacA* s, one (Italy 23) showed the *vacA* m2 genotype (*cagA*-positive); another (Italy 24, *cagA*-positive) contained two different *vacA* m1 sequences, suggesting double infection; the third (Italy 36), undetermined for *vacA* s and m, harbored different *cagA* genotypes, also pointing to mixed infection. Of the 29 isolates with a definable *vacA* s1 genotype (that also included multiple- and single-strain infections), 12 (41%) were s1a, 14 (48%) s1b, and 3 (10%) were s1a and s1b recombinants (Table S2). The *vacA* s2 genotype was less frequent (5/34 cases, 15%) and mixed infection was found in 1/5 positive cases.

With regard to the m region, 16/34 cases (47%) had the m1 allele (including two mixed); the m2 was also found in 16/34 cases (47%, including two mixed). The remaining 3 cases (3/37, 6%) could not be subtyped for the m region.

Thus, excluding five isolates where both the signal and the middle regions were undetectable, the following *vacA* type combinations were identified in the NNGDLs: s1a-m1 (7/32, 22%), s1a-m2 (3/32, 9%), s1b-m1 (5/32, 16%), s1b-m2 (7/32, 22%), s1b-m1/m2 (2/32, 6%), rec s1a/s1b-m1 (3/32, 9%), and s2-m2 (5/32, 16%).

### 3.3. Comparison between the Hp Genotypes of GINs and NNGDLs

The *cagA* gene was detected in 4/9 GINs and 29/37 NNGDLs, with an overall prevalence of 72% (33/46) among all the *Hp*-positive GINs and NNGDLs. The *vacA* s1a and m1 subtypes were much more frequent in the *vacA*-positive GINs than in the *vacA*-positive NNGDLs (6/7 vs. 12/34 for s1a, p=0.014; 7/7 vs. 18/34 for m1, p=0.020), while the s1b subtype, not found in the 7 *vacA*-positive GINs, was detected in 14/34 *vacA*-positive NNGDLs (0/7 vs. 14/34, p=0.036).

Most of the retrieved *Hp* sequences were unique, with an excess of gene diversity (H=1.0000+/-0.0047) for *vacA*, higher for *vacA* s (0.9838+/-0.0086) compared to *vacA* m (0.9212+/-0.0241). A driving force that could underlie such high levels of sequence variation is population expansion under neutral evolution. To highlight the deviation of the sequence variability pattern from neutral expectations, we performed a Fu’s Fs neutrality test on all the *vacA* sequences and, separately, on the s and m regions. A largely negative test value (Fs=-21.28254; p<0.000001) pointed to a deviation from neutral expectation for *vacA*. Fu’s Fs-test indicated an excess of recent mutations for the s region, supported by a significant negative value (Fs=-7.23602; p=0.041) and selective neutrality for the m region.

**Figure 1:**
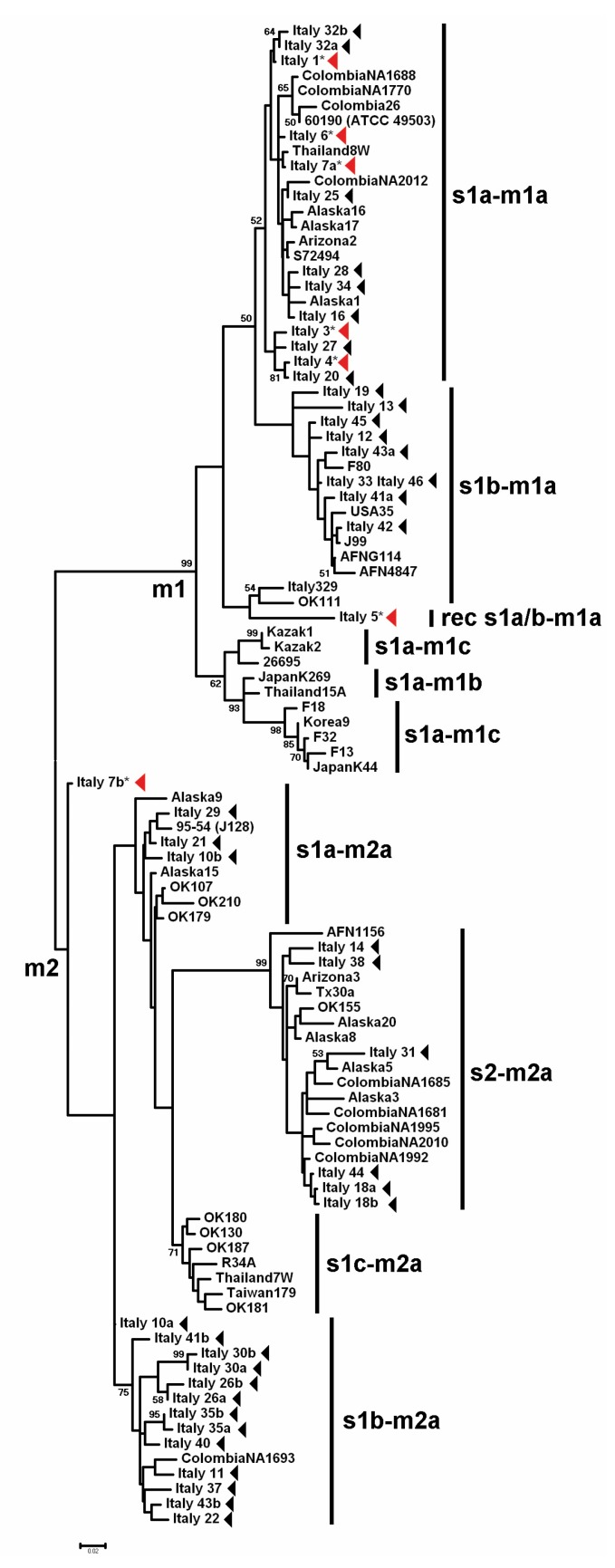
Phylogenetic tree of 100 *vacA* s and m *Helicobacter pylori* sequences. The 45 sequences obtained in this study are indicated by arrowheads; asterisks and red arrowheads highlight association with gastric intraepithelial neoplasia. Other sequences, labeled according to strain designation, are from GenBank.

**Figure 2:**
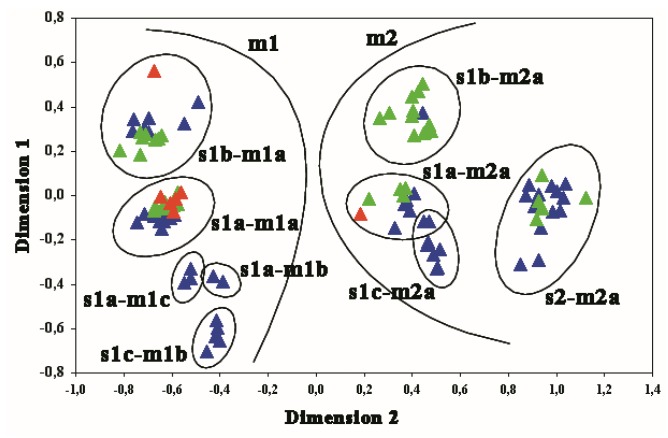
Multidimensional scaling plot of 45 *Helicobacter pylori vacA* s and m sequences obtained in this study (red: gastric intraepithelial neoplasia, GIN; green: non-neoplastic gastro-duodenal lesions, NNGDLs) and of 55 worldwide *Helicobacter pylori* sequences from GenBank (blue), based on the *vacA* gene (both s and m regions). Scaled stress value=0.0208.

To investigate the relationships between the *Hp* genotypes and the type of gastric lesion, we focused on 45 *vacA* s and m sequences from the present study (7 from GINs, 38 from NNGDLs) and on 55 worldwide GenBank sequences. The phylogenetic tree (Figure 1) and the MDS plot of the genotypes distance matrix showed that the *vacA* s and m sequences from the NNGDLs were distributed in 6/10 clusters (Figure 2), while almost all the GIN sequences clustered together in clade s1-m1. Phylogenetic analysis indicated a clear separation of the m1 and m2 sequences in two clades, with the *vacA* s subclusters within these clades. Five of the 7 *vacA* s and m GIN sequences clustered in the major s1a-m1a clade, although in two different subclusters. As highlighted by branch length, Italy 5, the only s1b-m1a sequence associated with GIN, differed from the other s1b sequences in the phylogenetic tree. This was consistent with the fact that Italy 5 presented a C variant typical of s1a at nucleotide position 269, suggesting an origin through recombination between s1a and s1b. The *vacA* s and m sequences retrieved from Italy 7 clustered in a sister branch of the m2 clade (as s1a-m2). In contrast, the *vacA* s and m sequences from the NNGDLs were distributed in all clades, except the s1c-m1 and s1c-m2 clusters, that included only East Asian isolates. Notably, two possible recombinants with consistent variation at *vacA* s (Italy 13 and Italy 19) joined a separate branch in subcluster s1b-m1a.

AMOVA, applied to all the *Hp* genotypes found in the GINs and in the various NNGDL subsets (non-atrophic gastritis, non-atrophic gastritis associated with gastric erosions, and non-atrophic gastritis associated with duodenal ulcer), showed that “within-groups” variance was 97.49% for both *vacA* s and m, and 95.33% considering only *vacA* s. “Among groups” variance was 2.51% (Fst: 0.02512, p<0.000001) for *vacA* s and m combined, and 4.67% (Fst: 0.04674, p<0.000001) when only *vacA* s was considered. The genetic variance of *vacA* m was almost completely (98.4%) “within-groups” (Fst: 0.01604, p<0.000001).

**Figure 3:**
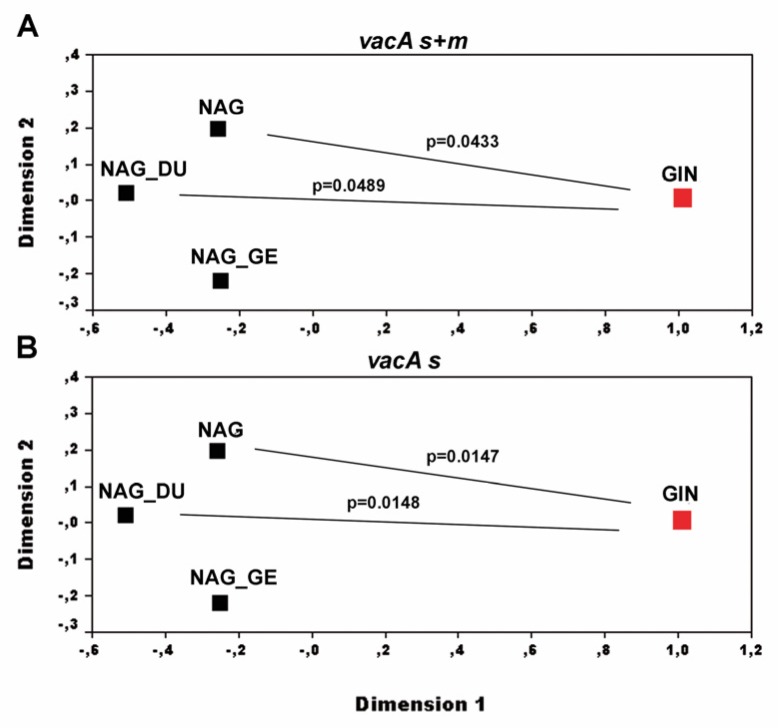
Multidimensional scaling plots of pairwise Fst values representing differences in *Helicobacter pylori* genotype distribution between gastric intraepithelial neoplasia (GIN, red square), non-atrophic antral gastritis alone (NAG), NAG plus duodenal ulcer (NAG_DU) and NAG plus gastric erosions (NAG_GE) (black squares). **A:** combined *vacA* s and m sequences. **B:** *vacA* s sequences.

Pairwise Fst values comparison, applied to the *vacA* sequences, highlighted a significant variance in the genotype distribution between the *Hp* sequences associated with GINs and NNGDLs. When Fst was computed on *vacA* s and m combined, the GIN genotypes significantly differed from those found in non-atrophic gastritis alone (Fst: 0.13618, p=0.04336±0.0021) and in non-atrophic gastritis plus duodenal ulcer (Fst: 0.18218, p=0.04891±0.0020) (Figure 3A). When Fst was computed on *vacA* s, the GIN genotypes significantly differed from those detected in non-atrophic gastritis alone (Fst: 0.17998, p=0.01485±0.0011) and in non-atrophic gastritis plus duodenal ulcer (Fst: 0.23198, p=0.01475±0.0012) (also with a trend for those associated with non-atrophic gastritis plus gastric erosions, Fst: 0.11173, p=0.06772±0.0028) (Figure 3B). Fst analysis did not show significant divergent variance in *vacA* m genotype distribution (not shown).

**Figure 4:**
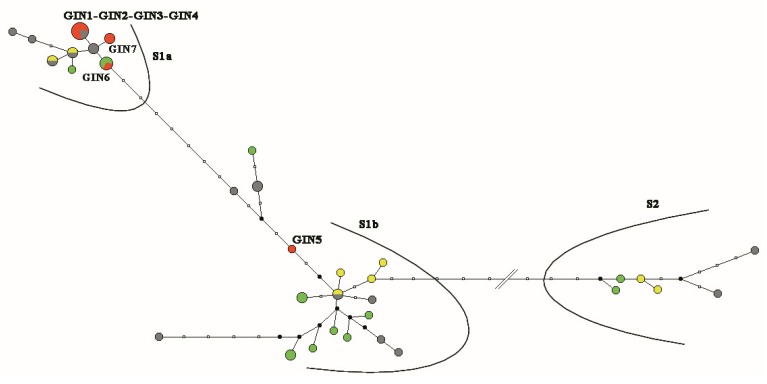
Median-joining (MJ) network of *vacA* s genotypes. The non-neoplastic gastro-duodenal lesions cluster to s1a, s1b, and s2, while the cases of gastric intraepithelial neoplasia (GIN), except GIN5 (Italy 5, an s1a/b recombinant), join cluster s1a. Dots representing gastric lesions are color-coded as follows: GINs, red; non-atrophic gastritis (NAG) alone, green; NAG plus gastric erosions, grey; NAG plus duodenal ulcer, yellow. Dot sizes reflect the number of cases sharing the same *vacA* s genotype.

The MJ network based on *vacA* s, used to visualize the relationships between the *Hp*-associated lesions, showed that the NNGDL genotypes joined clusters s1a, s1b, and s2, while the GIN genotypes clustered in s1a (Figure 4), except one s1a/b recombinant (Italy 5, Table S2). Duodenal ulcer was associated with the s1a genotype only in cases manifesting non-atrophic gastritis with gastric erosions, i.e., a more aggressive clinical phenotype (Italy 27 and Italy 28, Table S2).

**Figure 5:**
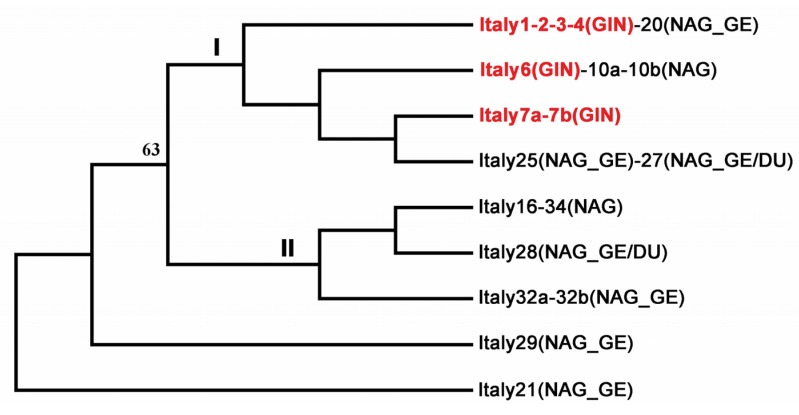
Maximum parsimony (MP) tree of the *vacA* s1a genotypes found in the present study. The MP method split the tree into two subclades, designated I and II (bootstrap value: 63%), highlighting a subclustering structure of the *vacA* s1a cluster. All the GIN-associated *Helicobacter pylori* sequences (red) were included in subclade I of clade s1a, suggesting strict evolutionary relationships. The MP tree was obtained using the Close-Neighbor-Interchange algorithm with search level 3, in which initial trees are generated by random addition of sequences (100 replicates). The bootstrap consensus tree, inferred from 1000 replicates, represents nine genotypes found in the *Helicobacter pylori* strains retrieved from 20 gastro-duodenal lesions. Multiple infections showing the same *vacA* s1a sequence are indicated with "a" and "b" in the strain designation. Concomitant lesions (non-atrophic gastritis alone (NAG), NAG plus duodenal ulcer (NAG_DU), and NAG plus gastric erosions (NAG_GE) are in brackets.

The subclustering of the MP tree for the s1a genotypes highlights a strict evolutionary relationship between the GIN *Hp* genotypes (Figure 5). The *vacA* s1a sequences were grouped based on four evolutionary parsimony informative sites of the cluster s1a dataset, one of which split the tree into two subclades, s1aI and s1aII, with a significant 63% bootstrap value. The s1aI subclade included Italy 25 (non-atrophic gastritis plus gastric erosions), Italy 27 (non-atrophic gastritis plus duodenal ulcer), and all the GIN genotypes in the dataset. Two GINs shared *Hp* genotype with a case of non-atrophic gastritis plus gastric erosions (Italy 20), and with a case of non-atrophic gastritis alone (Italy 10). Subclade s1aII included sequences associated with non-atrophic gastritis alone, such as Italy 16 and 34 (same genotype) and Italy 32, and non-atrophic gastritis plus duodenal ulcer (Italy 28). Italy 21 and Italy 29 (both non-atrophic gastritis plus gastric erosions) were separated by distance from subclades s1aI and s1aII.

The *Hp vacA* s1a genotype was associated with the NNGDLs in 35.3% (12/34) of the cases. In contrast, 86% (6/7) of the *Hp*-positive GINs had the *vacA* s1a genotype. Although confidence intervals were unreliable due to small numbers, the OR computed comparing GINs and NNGDLs (OR, 11; 95% CI 1.2-99.4; χ2: 6.193, p=0.012) suggests an association between the *vacA* s1a *Hp* genotype and GC risk.

Finally, the *cagA* 5’ sequences detected in our study were closely related to a cluster containing only Western strains (data not shown), which is consistent with evidence that this region may distinguish European from East Asian *Hp* genotypes [48].

## 4. Discussion

The present study analyzed a series of GINs and NNGDLs from a high-GC-risk area in Northern Italy [16] for genetic evidence of *Hp* infection and for *Hp* genotype characteristics in the *cagA* 5’ and *vacA* s and m regions. We found *Hp* sequences in only 27% of the GIN cases. This confirms that *Hp* can be retrieved from GINs, although it is well known that infection with this pathogen, which is the strongest known non-cardia GC risk factor, is lost during the neoplastic progression to GC [18]. Given the relatively high frequency of *Hp* infection in Italy [12,13], the finding of *Hp* in GIN does not necessarily imply that infection contributed to the pathogenesis of the lesion. However, the GIN-associated *Hp* strains found in this study tended to contain the *cagA* pathogenicity island and pertained to the *vacA* s1 and m1 genotypes, known to be associated with severe disease [5,11]. This suggests a specific selection of aggressive strains, which could contribute to GIN.

It has also been demonstrated that distinct *Hp vacA* s genotypes have preferential geographical distributions [11,49,50,51,52]. Few Italian studies reported the results of *Hp vacA* genotyping in gastric lesions. Two studies on dyspeptic patients from Central and Southern Italy [20,24] found a higher prevalence of *vacA* s1 compared to s2. Furthermore, in Northeast Italy, Zambon et al. [22] reported that the *cagA*-positive *vacA* s1 genotype was more common in patients with gastric ulcer or duodenitis than in patients with antral-predominant gastritis only. We confirmed the high prevalence of *vacA* s1 in the cases tested (GINs and NNGDLs) and, after characterization of the *vacA* subtype, we found that 44% of the cases were s1a, 34% s1b, 10% s1a/s1b recombinants, and the remaining 12% s2.

Various studies linked the gastric disease phenotype to the *vacA* genotype of the infecting *Hp* strain. The *vacA* s1-m1 strain is highly cytotoxic [5,10] and is often detected in patients with precancerous lesions [10,53,54]. In our study, all the GIN-associated strains showed the *vacA* s1-m1 genotype, and, except for one s1a/s1b recombinant, were of s1a subtype. In contrast, the *Hp* strains retrieved from the NNGDLs showed no significant difference in s1a and s1b frequencies.

The conventional Fst approach confirmed the importance of the discrepancy in *vacA* s frequencies, showing that the variance of *vacA* genotypes distribution in the GINs significantly differed from that in the NNGDLs, particularly non-atrophic antral gastritis and non-atrophic antral gastritis plus duodenal ulcer, and that *vacA* m, despite its reportedly independent association with cytotoxic activity [10], was not related to the GINs. In fact, network analysis based on *vacA* s genotypes showed that all the GIN-associated *Hp* sequences, except an s1a/b recombinant, were in cluster s1a. Moreover, the MP method, used to verify the subclustering structure of cluster s1a, highlighted that the *Hp* genotypes associated with the GINs were strictly evolutionarily related in the s1aI subclade. Thus, while our study design does not allow an accurate estimate of the GC risk conferred by *Hp* infection, the observed OR value suggests that infection with locally-circulating *Hp vacA* s1a strains might contribute to high GC risk in the Cremona area.

## 5. Conclusions

*Hp* is a highly heterogeneous pathogen and the *Hp* strains circulating in Italy remain poorly known. The genetic heterogeneity observed in this study, focused on a high-GC-risk area, could reflect a sudden expansion of *Hp* strains, as evidenced by the Fu’s Fs-test, indicating an excess of recent mutations. The GIN-associated *Hp* sequences tended to cluster together, suggesting strict evolutionary relationships, consistent with the fact that the cases examined derive from a restricted geographic region [16], where GC risk could be contributed by pathogenic strains that evolved locally from a unique founder. Our study has some limitations, including small sample size, focus on a limited geographic area, and lack of documentation of other factors, such as tobacco use, that could influence GC risk. This should be clarified by additional investigations involving larger numbers of GINs/GCs and NNGDLs from geographic areas with different levels of risk in Italy and elsewhere.

## Supplementary Materials

The following are available online at https://www.mdpi.com/2076-2607/8/3/324/s1: **Table S1:** PCR primers used to amplify the human β-globin gene and the *Helicobacter pylori glmM*, *cagA,* and *vacA* sequences; **Table S2:** Pathological characteristics of the 46 *H. pylori*-positive cases studied with genotyping results.

## References

[1] Polk D.B., Peek R.M. (2010). Helicobacter pylori: Gastric cancer and beyond. Nat. Rev. Cancer.

[2] Parsonnet J., Friedman G.D., Vandersteen D.P., Chang Y., Vogelman J.H., Orentreich N., Sibley R.K. (1991). Helicobacter pylori infection and the risk of gastric carcinoma. N. Engl. J. Med..

[3] IARC Working Group on the Evaluation of Carcinogenic Risks to Humans (2012). Biological Agents. A Review of Human Carcinogens.

[4] Lyons K., Le L.C., Pham Y.T., Borron C., Park J.Y., Tran C.T.D., Tran T.V., Tran H.T., Vu K.T., Do C.D. (2019). Gastric cancer: Epidemiology, biology, and prevention: A mini review. Eur. J. Cancer Prev. Off. J. Eur. Cancer Prev. Organ..

[5] Cover T.L. (2016). Helicobacter pylori diversity and gastric cancer risk. MBio.

[6] Censini S., Lange C., Xiang Z., Crabtree J.E., Ghiara P., Borodovsky M., Rappuoli R., Covacci A. (1996). Cag, a pathogenicity island of Helicobacter pylori, encodes type I-specific and disease-associated virulence factors. Proc. Natl. Acad. Sci. USA.

[7] Covacci A., Censini S., Bugnoli M., Petracca R., Burroni D., Macchia G., Massone A., Papini E., Xiang Z., Figura N. (1993). Molecular characterization of the 128-kDa immunodominant antigen of Helicobacter pylori associated with cytotoxicity and duodenal ulcer. Proc. Natl. Acad. Sci. USA.

[8] Kuck D., Kolmerer B., Iking-Konert C., Krammer P.H., Stremmel W., Rudi J. (2001). Vacuolating cytotoxin of Helicobacter pylori induces apoptosis in the human gastric epithelial cell line AGS. Infect. Immun..

[9] Galmiche A., Rassow J., Doye A., Cagnol S., Chambard J.C., Contamin S., de Thillot V., Just I., Ricci V., Solcia E. (2000). The N-terminal 34 kDa fragment of Helicobacter pylori vacuolating cytotoxin targets mitochondria and induces cytochrome c release. EMBO J..

[10] Atherton J.C., Cao P., Peek R.M., Tummuru M.K., Blaser M.J., Cover T.L. (1995). Mosaicism in vacuolating cytotoxin alleles of Helicobacter pylori. Association of specific vacA types with cytotoxin production and peptic ulceration. J. Biol. Chem..

[11] Atherton J.C., Peek R.M., Tham K.T., Cover T.L., Blaser M.J. (1997). Clinical and pathological importance of heterogeneity in vacA, the vacuolating cytotoxin gene of Helicobacter pylori. Gastroenterology.

[12] Palli D., Vaira D., Menegatti M., Saieva C. (1997). A serologic survey of Helicobacter pylori infection in 3281 Italian patients endoscoped for upper gastrointestinal symptoms. The Italian Helicobacter Pylori Study Group. Aliment. Pharmacol. Ther..

[13] Gasbarrini A., Anti M., Franceschi F., Armuzzi A., Cotichini R., Ojetti V., Candelli M., Lippi M.E., Paolucci M., Cicconi V. (2001). Prevalence of and risk factors for Helicobacter pylori infection among healthcare workers at a teaching hospital in Rome: The Catholic University Epidemiological Study. Eur. J. Gastroenterol. Hepatol..

[14] Zamani M., Ebrahimtabar F., Zamani V., Miller W.H., Alizadeh-Navaei R., Shokri-Shirvani J., Derakhshan M.H. (2018). Systematic review with meta-analysis: The worldwide prevalence of Helicobacter pylori infection. Aliment. Pharmacol. Ther..

[15] Toracchio S., Marzio L. (2003). Primary and secondary antibiotic resistance of Helicobacter pylori strains isolated in central Italy during the years 1998-2002. Dig. Liver Dis. Off. J. Ital. Soc. Gastroenterol. Ital. Assoc. Study Liver.

[16] Capocaccia R., De Angelis R., Frova L., Sant M., Buiatti E., Gatta G., Micheli A., Berrino F., Barchielli A., Conti E. (1995). Estimation and projections of stomach cancer trends in Italy. Cancer Causes Control CCC.

[17] Ferlay J., Autier P., Boniol M., Heanue M., Colombet M., Boyle P. (2007). Estimates of the cancer incidence and mortality in Europe in 2006. Ann. Oncol. Off. J. Eur. Soc. Med Oncol..

[18] Forman D.B.F., Brewster D.H., Gombe Mbalawa C., Kohler B., Piñeros M., Steliarova-Foucher E., Swaminathan R., Ferlay J. (2014). Cancer Incidence in Five Continents. Cancer Incidence in Five Continents.

[19] Stroffolini T., Rosmini F., Ferrigno L., Fortini M., D’Amelio R., Matricardi P.M. (1998). Prevalence of Helicobacter pylori infection in a cohort of Italian military students. Epidemiol. Infect..

[20] Gallo N., Zambon C.F., Navaglia F., Basso D., Guariso G., Grazia Piva M., Greco E., Mazza S., Fogar P., Rugge M. (2003). Helicobacter pylori infection in children and adults: A single pathogen but a different pathology. Helicobacter.

[21] Russo F., Berloco P., Cuomo R., Caruso M.L., Di Matteo G., Giorgio P., De Francesco V., Di Leo A., Ierardi E. (2003). Helicobacter pylori strains and histologically-related lesions affect the outcome of triple eradication therapy: A study from southern Italy. Aliment. Pharmacol. Ther..

[22] Zambon C.F., Navaglia F., Basso D., Rugge M., Plebani M. (2003). Helicobacter pylori babA2, cagA, and s1 vacA genes work synergistically in causing intestinal metaplasia. J. Clin. Pathol..

[23] Figura N., Valassina M., Roviello F., Pinto F., Lenzi C., Giannace R., Marrelli D., Valentini M., Valensin P.E. (2000). Helicobacter pylori cagA and vacA types and gastric carcinoma. Dig. Liver Dis. Off. J. Ital. Soc. Gastroenterol. Ital. Assoc. Study Liver.

[24] Cellini L., Grande R., Di Campli E., Di Bartolomeo S., Capodicasa S., Marzio L. (2006). Analysis of genetic variability, antimicrobial susceptibility and virulence markers in Helicobacter pylori identified in Central Italy. Scand. J. Gastroenterol..

[25] De Francesco V., Margiotta M., Zullo A., Hassan C., Valle N.D., Burattini O., D’Angelo R., Stoppino G., Cea U., Giorgio F. (2006). Claritromycin resistance and Helicobacter pylori genotypes in Italy. J. Microbiol..

[26] Basso D., Zambon C.F., Letley D.P., Stranges A., Marchet A., Rhead J.L., Schiavon S., Guariso G., Ceroti M., Nitti D. (2008). Clinical relevance of Helicobacter pylori cagA and vacA gene polymorphisms. Gastroenterology.

[27] Grande R., Di Campli E., Di Bartolomeo S., Verginelli F., Di Giulio M., Baffoni M., Bessa L.J., Cellini L. (2012). Helicobacter pylori biofilm: A protective environment for bacterial recombination. J. Appl. Microbiol..

[28] Donida B.M., Tomasello G., Ghidini M., Buffoli F., Grassi M., Liguigli W., Maglietta G., Pergola L., Ratti M., Sabadini G. (2019). Epidemiological, clinical and pathological characteristics of gastric neoplasms in the province of Cremona: The experience of the first population-based specialized gastric cancer registry in Italy. BMC Cancer.

[29] McColl K.E., el-Omar E., Gillen D. (2000). Helicobacter pylori gastritis and gastric physiology. Gastroenterol. Clin. North Am..

[30] Schlemper R.J., Riddell R.H., Kato Y., Borchard F., Cooper H.S., Dawsey S.M., Dixon M.F., Fenoglio-Preiser C.M., Flejou J.F., Geboes K. (2000). The Vienna classification of gastrointestinal epithelial neoplasia. Gut.

[31] Kato M. (2015). Diagnosis and therapies for gastric non-invasive neoplasia. World J. Gastroenterol..

[32] Dixon M.F., Genta R.M., Yardley J.H., Correa P. (1996). Classification and grading of gastritis. The updated Sydney System. International Workshop on the Histopathology of Gastritis, Houston 1994. Am. J. Surg. Pathol..

[33] Linee Guida (2015). Tracciabilità, Raccolta, Trasporto, Conservazione e Archiviazione Di Cellule e Tessuti Per Indagini Diagnostiche Di Anatomia Patologica. http://www.salute.gov.it/imgs/C_17_pubblicazioni_2369_allegato.pdf.

[34] Rigoli L., Di Bella C., Verginelli F., Falchetti M., Bersiga A., Rocco A., Nardone G., Mariani-Costantini R., Caruso R.A. (2008). Histological heterogeneity and somatic mtDNA mutations in gastric intraepithelial neoplasia. Mod. Pathol. Off. J. USA Can. Acad. Pathol..

[35] Rocco A., Caruso R., Toracchio S., Rigoli L., Verginelli F., Catalano T., Neri M., Curia M.C., Ottini L., Agnese V. (2006). Gastric adenomas: Relationship between clinicopathological findings, Helicobacter pylori infection, APC mutations and COX-2 expression. Ann. Oncol. Off. J. Eur. Soc. Med Oncol..

[36] Kumar S., Stecher G., Li M., Knyaz C., Tamura K. (2018). MEGA X: Molecular evolutionary genetics analysis across computing platforms. Mol. Biol. Evol..

[37] Kimura M. (1980). A simple method for estimating evolutionary rates of base substitutions through comparative studies of nucleotide sequences. J. Mol. Evol..

[38] Wright S. (1951). The genetical structure of populations. Ann. Eugen..

[39] Excoffier L., Lischer H.E. (2010). Arlequin suite ver 3.5: A new series of programs to perform population genetics analyses under Linux and Windows. Mol. Ecol. Resour..

[40] Rozas J., Ferrer-Mata A., Sanchez-DelBarrio J.C., Guirao-Rico S., Librado P., Ramos-Onsins S.E., Sanchez-Gracia A. (2017). DnaSP 6: DNA sequence polymorphism analysis of large data sets. Mol. Biol. Evol..

[41] Bandelt H.J., Forster P., Rohl A. (1999). Median-joining networks for inferring intraspecific phylogenies. Mol. Biol. Evol..

[42] Fu Y.X. (1997). Statistical tests of neutrality of mutations against population growth, hitchhiking and background selection. Genetics.

[43] Polzin T.D., Daneshmand S.V. (2003). On Steiner trees and minimum spanning trees in hypergraphs. Oper. Res. Lett..

[44] Felsenstein J. (1985). Confidence limits on phylogenies: An approach using the bootstrap. Evol. Int. J. Org. Evol..

[45] Nei M., Kumar S. (2000). Molecular Evolution and Phylogenetics.

[46] Thompson J.D., Higgins D.G., Gibson T.J. (1994). CLUSTAL W: Improving the sensitivity of progressive multiple sequence alignment through sequence weighting, position-specific gap penalties and weight matrix choice. Nucleic Acids Res..

[47] Hatakeyama M. (2014). Helicobacter pylori CagA and gastric cancer: A paradigm for hit-and-run carcinogenesis. Cell Host Microbe.

[48] Yamaoka Y., Orito E., Mizokami M., Gutierrez O., Saitou N., Kodama T., Osato M.S., Kim J.G., Ramirez F.C., Mahachai V. (2002). Helicobacter pylori in North and South America before Columbus. FEBS Lett..

[49] Covacci A., Telford J.L., Del Giudice G., Parsonnet J., Rappuoli R. (1999). Helicobacter pylori virulence and genetic geography. Science.

[50] Kersulyte D., Mukhopadhyay A.K., Velapatino B., Su W., Pan Z., Garcia C., Hernandez V., Valdez Y., Mistry R.S., Gilman R.H. (2000). Differences in genotypes of Helicobacter pylori from different human populations. J. Bacteriol..

[51] Van Doorn L.J., Figueiredo C., Megraud F., Pena S., Midolo P., Queiroz D.M., Carneiro F., Vanderborght B., Pegado M.D., Sanna R. (1999). Geographic distribution of vacA allelic types of Helicobacter pylori. Gastroenterology.

[52] Yamaoka Y., Kodama T., Gutierrez O., Kim J.G., Kashima K., Graham D.Y. (1999). Relationship between Helicobacter pylori iceA, cagA, and vacA status and clinical outcome: Studies in four different countries. J. Clin. Microbiol..

[53] Naumann M., Crabtree J.E. (2004). Helicobacter pylori-induced epithelial cell signalling in gastric carcinogenesis. Trends Microbiol..

[54] van Doorn L.J., Figueiredo C., Sanna R., Plaisier A., Schneeberger P., de Boer W., Quint W. (1998). Clinical relevance of the cagA, vacA, and iceA status of Helicobacter pylori. Gastroenterology.

