# Supplementary material for: Evolutionarily-Related Helicobacter pylori Genotypes and Gastric Intraepithelial Neoplasia in a High-Risk Area of Northern Italy
Source: Microorganisms. 2020 Feb 26;8(3):324. doi: 10.3390/microorganisms8030324 (PMC7142731; doi:10.3390/microorganisms8030324)
Supplement: Supplementary file 1 [file microorganisms-08-00324-s001.zip › Supplementary Tables Proof Read/Table S1.docx]

**Table S1** – PCR primers used to amplify human *β-globin* and the *Helicobacter pylori* *glmM*, *cagA* and *vacA* sequences.

***β-globin***

External PCR (172 bp)

S7-fwd 5’-GCTTCTGACACAACTGTGTTCAC-3’

A178-rev 5’-GTCTCCTTAAACCTGTCTTGTAACC-3’

Nested PCR (102 bp)

S22-fwd 5’-GTGTTCACTAGCAACCTCAAACAG-3’

A123-rev 5’-CAACTTCATCCACGTTCACC-3’

***glmM***

External PCR (144 bp)

F1 5’-TAACCGAAGACATGCGCTG-3’

R 5’-CATGAAAGATTTCTTCAATCAATCGCT-3’

Semi-nested PCR (137 bp)

F2 5’-AGACATGCGCTGTGATGC-3’

R 5’-CATGAAAGATTTCTTCAATCAATCGCT-3’

***cagA* 5’ region**

External PCR (158 bp)

F1 5’-CAATCGTTGATAAGAAYGATAGGG-3’

R1 5’-GTCTTTGTTGATYAAATCATTGCTC-3’

Nested PCR (148 bp)

F2 5’-GATAAGAAYGATAGGGATAAYAGGC-3’

R2 5’-CTTTGTTGATYAAATCATTGCTC-3’

***vacA* s1/s2**

External PCR [s1 (164 bp)/s2 (193 bp)]

F 5’-ACACACCGCAAAATCAATCG-3’

R1 5’-AGCCCTGARACCGTTCCTACAG-3’

Semi-nested PCR [s1 (122 bp)/s2 (151 bp)]

F 5’-ACACACCGCAAAATCAATCG-3’

R2 5’-CCAACAATGGCTGGAATRATCA-3’C

***vacA* m1/m2**

External PCR [m1 (153 bp)/m2 (168 bp)]

mF1 5’-ACCGCTCATBAAGATYAAYARCGCTC-3’

mR1 5’-GCTAGGCGCTCTTTGAATTGC-3’

Semi-nested PCR [m1 (151 bp)/m2 (166 bp)]

mF1 5’-ACCGCTCATBAAGATYAAYARCGCTC-3’

mR2 5’-TAGGCGCTCTTTGAATTGCTC-3’
