# Supplementary material for: Evolutionarily-Related Helicobacter pylori Genotypes and Gastric Intraepithelial Neoplasia in a High-Risk Area of Northern Italy
Source: Microorganisms. 2020 Feb 26;8(3):324. doi: 10.3390/microorganisms8030324 (PMC7142731; doi:10.3390/microorganisms8030324)
Supplement: Supplementary file 1 [file microorganisms-08-00324-s001.zip › Supplementary Tables Proof Read/Table S2.doc]

**Table S2** – Pathological characteristics of the 46 *Helicobacter pylori*-positive cases and genotyping results.

| Cases | Pathological diagnosis | ***vacA* s** | ***vacA* m** | ***cagA*** |
| --- | --- | --- | --- | --- |
| Italy 1 | Gastric intraepithelial neoplasia | s1a | m1 | + |
| Italy 2 | Gastric intraepithelial neoplasia | s1a | *NA* | - |
| Italy 3 | Gastric intraepithelial neoplasia | s1a | m1 | + |
| Italy 4 | Gastric intraepithelial neoplasia /gastric cancer | s1a | m1 | + |
| Italy 5 | Gastric intraepithelial neoplasia /gastric cancer | rec s1a/s1b | m1 | - |
| Italy 6 | Gastric intraepithelial neoplasia /gastric cancer | s1a | m1 | - |
| Italy 7 | Gastric intraepithelial neoplasia /gastric cancer | s1a | m1+m2 | +# |
| Italy 8 | Gastric intraepithelial neoplasia | *na*^°^ | m1 | - |
| Italy 9* | Gastric intraepithelial neoplasia | *Na* | *NA* | - |
| Italy 10 | Nonatrophic antral gastritis | s1a | m2# | - |
| Italy 11 | Nonatrophic antral gastritis | s1b | m2 | - |
| Italy 12 | Nonatrophic antral gastritis | s1b | m1 | + |
| Italy 13 | Nonatrophic antral gastritis | rec s1a/s1b | m1 | + |
| Italy 14 | Nonatrophic antral gastritis with gastric erosions | s2 | m2 | - |
| Italy 15 | Nonatrophic antral gastritis | s1a | *NA* | + |
| Italy 16 | Nonatrophic antral gastritis | s1a | m1 | - |
| Italy 17 | Nonatrophic antral gastritis with gastric erosions | rec s1a/s1b# | m1 | + |
| Italy 18 | Nonatrophic antral gastritis with duodenal ulcer | s2# | m2 | - |
| Italy 19 | Nonatrophic antral gastritis with gastric erosions | rec s1a/s1b | m1 | + |
| Italy 20 | Nonatrophic antral gastritis with gastric erosions | s1a | m1 | + |
| Italy 21 | Nonatrophic antral gastritis with gastric erosions | s1a | m2 | - |
| Italy 22 | Nonatrophic antral gastritis with gastric erosions | s1b | m2 | +# |
| Italy 23 | Nonatrophic antral gastritis with gastric erosions | *NA* | m2 | + |
| Italy 24 | Nonatrophic antral gastritis | *NA* | m1# | + |
| Italy 25 | Nonatrophic antral gastritis with gastric erosions | s1a | m1 | +# |
| Italy 26 | Nonatrophic antral gastritis with gastric erosions | s1b | m2# | + |
| Italy 27 | Nonatrophic antral gastritis with gastric erosions and duodenal ulcer | s1a | m1 | + |
| Italy 28 | Nonatrophic antral gastritis with gastric erosions and duodenal ulcer | s1a | m1 | +# |
| Italy 29 | Nonatrophic antral gastritis with gastric erosions | s1a | m2 | + |
| Italy 30 | Nonatrophic antral gastritis with gastric erosions | s1b# | m2 | + |
| Italy 31 | Nonatrophic antral gastritis | s2 | m2 | - |
| Italy 32 | Nonatrophic antral gastritis | s1a | m1# | + |
| Italy 33 | Nonatrophic antral gastritis with gastric erosions | s1b | m1 | +# |
| Italy 34 | Nonatrophic antral gastritis with gastric erosions | s1a | m1 | + |
| Italy 35 | Nonatrophic antral gastritis with duodenal ulcer | s1b | m2 | + |
| Italy 36 | Nonatrophic antral gastritis | *NA* | *NA* | +# |
| Italy 37 | Nonatrophic antral gastritis | s1b | m2 | - |
| Italy 38 | Nonatrophic antral gastritis with gastric erosions | s2 | m2 | + |
| Italy 39 | Nonatrophic antral gastritis with gastric erosions | s1a | *NA* | + |
| Italy 40 | Nonatrophic antral gastritis with duodenal ulcer | s1b | m2 | + |
| Italy 41 | Nonatrophic antral gastritis | s1b | m1+m2# | + |
| Italy 42 | Nonatrophic antral gastritis with duodenal ulcer | s1b | m1 | + |
| Italy 43 | Nonatrophic antral gastritis | s1b | m1+m2# | + |
| Italy 44 | Nonatrophic antral gastritis | s2 | m2 | +# |
| Italy 45 | Nonatrophic antral gastritis | s1b | m1 | + |
| Italy 46 | Nonatrophic antral gastritis with duodenal ulcer | s1b | m1 | +# |

*: Positive for the *glmM* (ureC) gene only; *NA*: not amplifiable; #: infection with multiple *H. pylori* strains
